# Diversity in Surface Microstructures of Trichomes, Epidermal Cells, and Stomata in Lentil Germplasm

**DOI:** 10.3389/fpls.2021.697692

**Published:** 2021-07-12

**Authors:** Ishita Patel, Linda Yuya Gorim, Karen Tanino, Albert Vandenberg

**Affiliations:** ^1^Department of Plant Sciences, College of Agriculture and Bioresources, University of Saskatchewan, Saskatoon, SK, Canada; ^2^Department of Agricultural, Food and Nutritional Science, Faculty of Agricultural, Life and Environmental Sciences, University of Alberta, Edmonton, AB, Canada

**Keywords:** lentil genotypes, trichomes, stomatal index, epidermal cells, wild lentil

## Abstract

To develop crops capable of withstanding challenges posed by climate change, breeding strategies must focus on addressing multiple stresses occurring concurrently in plants. Leaf epidermal structures such as trichomes, stomata, and epidermal cells play an important role in mediating plant defense and could be essential traits that impart wide-ranging tolerance to biotic and abiotic stresses. Consequently, it is important to inform on the underlying diversity in these traits in lentil germplasm (*Lens* spp.). In this study, we characterized foliar microstructures of 12 genotypes belonging to seven wild and cultivated *Lens* species. We performed scanning electron microscopy on leaflet and pod surfaces for their qualitative characterization. For quantitative characterization, we observed surface imprints *via* light microscopy and quantified trichome density (TD), trichome length (TL), stomatal density (SD), epidermal cell density (ECD), and stomatal index (SI) on adaxial and abaxial leaflet surfaces for each genotype. We also assessed the heritability of trichome traits by evaluating interspecific recombinant inbred lines (RILs) derived from the cross *Lens culinaris* CDC Redberry × *Lens tomentosus* IG 72805. Comparing foliar microstructures, we found that TD and TL varied widely among cultivated and wild lentil genotypes. However, in most lentil genotypes, the adaxial leaflet surface had lower TD and longer trichomes compared to the abaxial surface. Pubescence on pods comprised five major phenotypes: no trichomes or glabrous pods, very short trichomes at low density, short trichomes at high density, medium-length trichomes at high density, and long trichomes at high density. Leaves of all species were amphistomatous, and SI, SD, and ECD were all higher on the adaxial compared to the abaxial surface. Adaxial surfaces had slightly sunken stomata, which might be an adaptive trait to conserve water. Quantifying TD and TL on the leaflets of interspecific RILs revealed transgressive segregation of these traits, suggesting that TD and TL are quantitative in nature. While taxonomic implications of this study are limited, a detailed description of agronomically relevant morphophysiological traits presented in this paper along with the mode of inheritance of trichomes may serve as a resource for scientists developing lentil adapted to concurrent biotic and abiotic stresses of the future.

## Introduction

Lentil (*Lens culinaris* subsp. *culinaris* Medikus) is a globally important crop due to its economic, ecological, and nutritive qualities. Worldwide, lentil production exceeds 5.8 million tons, and lentil is cultivated for domestic use and export in 44 countries with various climates spanning six continents (FAO, 2021). Lentil is a legume crop capable of biological nitrogen fixation, which reduces N fertilizer use and increases the yield of subsequent crops (Migliozzi et al., 2015; Niu et al., 2017). The inclusion of lentil in oilseed–cereal rotation systems has also been demonstrated to increase the crop productivity and stabilize yields in changing environments (Liu et al., 2019). Globally, lentil consumption is increasing four to five times more rapidly than other pulse crops because of its relative ease of dehulling and its rapid cooking time (Khazaei et al., 2019). Lentil is relatively inexpensive, packed with essential amino acids, vitamins, and minerals, and has long been consumed in Mediterranean, Middle Eastern, and Afro-Asian cultures (Iqbal et al., 2006; Yadav et al., 2007). Its role as a potential replacement for animal-sourced protein is now being evaluated as a part of a global shift toward environmentally sustainable food systems (Jarpa-Parra, 2018; Warne et al., 2019).

Genus *Lens* (Miller) of the family Leguminosae consists of seven taxa that were classified into four species based on morphological and genomic differences (Ferguson et al., 2000): the cultivated lentil, *L. culinaris* subsp. *culinaris*, and six wild lentil taxa, *L. culinaris* subsp. *orientalis, L. culinaris* subsp. *tomentosus* (Ladizinsky) Ferguson, Maxted, van Slageren and Robertson, *L. culinaris* subsp. *odemensis* (Ladizinsky) Ferguson, Maxted, van Slageren and Robertson, *L. lamottei* Czefr., *L. ervoides* (Brign.) Grande, and *L. nigricans* (M. Bieb.) Godron. Most recently, based on genotyping-by-sequencing, Wong et al. (2015) suggested that the seven *Lens* taxa should be separated into four gene pools based on their ability to produce viable seeds after hybridization with cultivated lentil. They found *L. odemensis* to be distinct from *L. culinaris* and suggested that it should be a separate species instead of being a *L. culinaris* subspecies (Wong et al., 2015). According to the latest classification by Wong et al. (2015), the four gene pools are primary gene pool (*L. culinaris*, *L. orientalis*, and *L. tomentosus*), which have high interspecific crossability with each other; secondary gene pool (*L. lamottei* and *L. odemensis*); tertiary gene pool (*L. ervoides*); and quaternary gene pool (*L. nigricans*). Species in the same gene pool are reproductively compatible and produce viable seed, whereas species from different gene pools may not hybridize easily due to embryo abortion (Davies et al., 2007). *L. nigricans* (quaternary gene pool) is the most genetically distant from the cultivated lentil and has not been successfully crossed with *L. culinaris* (Ogutcen et al., 2018) or any other species.

Lentil is an annual, herbaceous, self-pollinating cool-season food legume with cleistogamous flowers and indeterminate growth habit (Smýkal et al., 2015). The lentil species are naturally distributed widely across diverse landscapes from the Western Mediterranean to Central Asia, an immense east–west region characterized physically by changes in elevation from sea level to high mountains at mid-latitudes. *L. orientalis* is considered the putative wild progenitor of cultivated lentil and was domesticated in the Fertile Crescent (South Eastern Turkey–Northern Syria) (Sonnante et al., 2009). *L. orientalis* is widely distributed along the steppes and dry areas of central and southwest Asia and from Turkey to Uzbekistan (Ladizinsky, 1979). Other wild lentil species have a wide, mostly Mediterranean geographical distribution, extending from Southern France to Spain to Morocco, and from Turkey, Syria, Iraq, to Uzbekistan (Smýkal et al., 2015). Wild lentil species are distributed across habitats ranging from open or partially shaded (*L. orientalis*, *L. tomentosus*, *L. odemensis*, and *L. nigricans*) to mostly shady, such as in pine forests (*L. ervoides*) (Ferguson and Erskine, 2001). The lentil species also exhibit winter and spring growth habit. It is presumed that during the domestication process, cultivated lentil went through population bottlenecks that narrowed their genetic base (Erskine et al., 1998). Its genetically accessible wild relatives have so far been mainly considered potential sources of genetic diversity and may be of value in breeding for resistance to biotic stresses, mostly for resistance to fungal plant pathogens (Coyne et al., 2020).

Increasing concerns about changes in climate have resulted not only in a shift toward plant-based foods, but also in additional interest in the capacity to breed food crops with greater resilience to climate change. It is becoming increasingly evident that global warming will not only result in an increase in biotic and abiotic stress in plants, but also unique combinations of two or more of these stresses (Pandey et al., 2017). Two of the major stresses of the future that are interlinked and interact with each other include increased pathogen stress and increased severity and frequency of drought (Szczepaniec and Finke, 2019). Recent studies of lentil species show that they employ various strategies to combat abiotic stresses, especially drought (Gorim and Vandenberg, 2017). Trichomes are distinctly more noticeable on *L. tomentosus*, which was also shown to transpire less water under severe drought stress (Gorim and Vandenberg, 2017, 2018). Various reviews shed light on the role of trichomes and stomata of plants in conferring tolerance to salt stress (Shabala, 2013), water stress (Arve et al., 2011), ozone stress (Oksanen, 2018), insect herbivory (Zavala et al., 2013; Kaur and Kariyat, 2020), pathogens (Melotto et al., 2008), and several other biotic and abiotic stresses (Karabourniotis et al., 2020). Epidermal cell size and number have also been shown to be affected by drought stress (McCree and Davis, 1974; Cutler et al., 1977; Bosabalidis and Kofidis, 2002; Lechner et al., 2008), root penetration stress (Beemster and Masle, 1996), and salt stress (Curtis and Läuchli, 1987). Genetic improvement strategies that include potential benefits of use of traits such as trichome morphology and stomatal characteristics may contribute to the development of lentil germplasm with increased stress tolerance. To date, however, there are no studies that characterize the trichomes, stomata, and epidermal cells of lentil species that could allow the development of breeding strategies to improve the resilience of the cultivated lentil.

In this study, we explored the existing diversity on the surfaces of leaflets and pods in wild and cultivated *Lens* spp. with respect to the traits of trichomes, stomata, and epidermal cells, with the goal that these morphophysiological traits will be useful to agronomists and breeders looking to identify or develop cultivars better suited to withstand a combination of biotic and abiotic stresses. We examined the foliar surfaces of 12 wild and cultivated lentil genotypes representative of the range of variation across the lentil species. We used the techniques of scanning electron microscopy and Suzuki’s Universal Micro-Printing (SUMP) method (Tanaka et al., 2005), which uses light microscopy, to visualize and characterize surface microstructures. Scanning electron microscopy has been previously used to study leaf epidermal traits to distinguish between and identify different taxa (Esfandani-Bozchaloyi and Zaman, 2018; Bahadur et al., 2019; Gul et al., 2019), and the SUMP method has been used in previous studies to visualize stomatal and epidermal cell anatomy and morphology (Tanaka et al., 2005; Arve et al., 2013; Banik et al., 2016; Carvalho et al., 2016). In this study, we used the imprints obtained from the SUMP method for quantitative characterization, while scanning electron microscopy was used for qualitative characterization of surface microstructural traits in leaflets and pods of *Lens* spp. While the literature exists on qualitative differences in leaf pubescence of cultivated lentil (Kumar and Solanki, 2014), this trait has not been quantitatively compared across wild and cultivated lentil species. Literature is also sparse on stomatal frequency, stomatal index (SI), and epidermal cell characteristics in lentil germplasm. These basic morphological traits can influence factors such as pathogen entry, insect herbivory, and water-use efficiency, which further influence seed yield and adaptation. Our study fills this gap in the literature and aims to quantitatively assess the variability across 12 wild and cultivated lentil genotypes. This study also quantifies the trichome density (TD) and trichome length (TL) in recombinant inbred lines (RILs) of the hybrid progeny of *L. culinaris* × *L. tomentosus*, informing on the inheritance of the trait of pubescence in lentil leaflets.

## Materials and Methods

### Plant Material and Experimental Design

The study was conducted under controlled conditions in the Phytotron facility of the College of Agriculture and Bioresources at the University of Saskatchewan, Saskatoon, Canada (52°07′58.8″N, 106°37′51.6″W). Table 1 shows the wild and cultivated lentil genotypes evaluated in the experiment. Prior to planting, all seeds were scarified, washed in bleach and Tween, and germinated in 50-ml Erlenmeyer flasks in the dark at 22°C. Germinated seedlings were transplanted into 9-L plastic pots filled with Sunshine Mix 4 (Sun Gro Horticulture, Canada) and kept in a Conviron GR48 growth chamber (Conviron, Winnipeg, Canada). About 500 mL of Hoagland solution was added to each pot before planting. The Hoagland solution contained 5 mM KNO_3_, 5 mM Ca(NO_3_)_2_, 2 mM MgSO_4_.7H_2_O, 2 mM KH_2_PO_4_, 45 μM Fe chelate (containing FeSO_4_.7H_2_O and EDTA 2Na.2H_2_O), and micronutrients (9.1 μM MnCl_2_.4H_2_O, 46.3 μM H_3_BO_3_, 0.76 μM ZnSO_4_.7H_2_O, 0.32 μM CuSO_4_.5H_2_O, and 0.1 μM Na_2_MoO_4_.2H_2_O).

**TABLE 1:** Species, genotype, center of origin, and gene pool of lentil genotypes used in the study.

**Species**	**Abbreviation**	**Genotype**	**Centre of origin^†^**	**Climate at center of origin**	**Gene pool**
*L. culinaris*	*L. cul.*	Indianhead	PI 320952 originating from Czechia (Carlisle, 2016)	Generally, temperature climate with increased precipitation and snowfall and decreasing annual average temperature as altitude increases (Dubrovsky et al., 2009). Mean annual precipitation ranges from 400 to 1,400 mm with majority of the areas receiving 600–800 mm (Beranová and Kyselý, 2018), and altitude ranges from 115 m to 1,602 m a.s.l. (Dubrovsky et al., 2009)	Primary
*L. culinaris*	*L. cul.*	CDC Redberry	Saskatoon, Canada	Semi-arid climate with short, warm, and dry summers with occasional rains and thunderstorms, and long, extreme winters with subzero temperatures (Mwakutuya, 2006). Altitude ranges from 436 to 1,067 m a.s.l. (Hopkins, 1938), and annual precipitation ranges from 300 to 430 mm (Mwakutuya, 2006)	Primary
*L. culinaris*	*L. cul.*	CDC Greenstar	Saskatoon, Canada	Same as CDC Redberry	Primary
*L. orientalis*	*L. ori.*	IG 72643	Latitude: 36.3375 Longitude: 36.8389 (Aleppo, Syria)	Mediterranean climate with hot, dry summers and cool, wet winters, with most rainfall occurring from September-October to April-May (Piggin et al., 2015). Hilly terrain, approximately 450 m above sea level (Google Maps, 2021). Mean temperature ranges from 5°C to 27°C, and the area receives an annual precipitation of 491 mm^†^	Primary
*L. orientalis*	*L. ori.*	PI 572376	Latitude: 37.67 Longitude: 29.13 (Denizli, Turkey)	Mountainous region with forested terrain at an elevation of 1,300 m above sea level (Google Maps, 2021). Mild and humid Mediterranean climate with warm winters averaging daily temperature of 6.7°C and hot summers with a mean daily temperature of 26.4°C (Gezer et al., 2014). Annual precipitation of 652 mm^†^	Primary
*L. tomentosus*	*L. tom.*	IG 72613	Latitude: 37.9167 Longitude: 40.2333 (Diyarbakir, Turkey)	Semi-arid climate with humid winters and dry summers, with most precipitation occurring in November to May (Gürsoy et al., 2014) and snow fall generally in December to February (Çaçan and Kökten, 2017). Average maximum temperature of 31°C and average minimum temperature of 1.8°C (Baran et al., 2011). Altitude of 670 m above sea level (Çaçan and Kökten, 2017) and annual precipitation of 521 mm^†^	Primary
*L. tomentosus*	*L. tom.*	IG 72614	Latitude: 37.9167 Longitude: 40.25 (Diyarbakir, Turkey)	Same as IG 72613	Primary
*L. tomentosus*	*L. tom.*	IG 72805	Latitude: 37.75 Longitude: 39.7667 (şanlıurfa, Turkey)	Arid region with hot, dry summers and cool, humid winters with average temperature in the summer and winter being 30–40°C and 5–10°C, respectively (Elmas, 1994; Ulukanligil et al., 2001). Elevation of 1,150 m a.s.l and annual precipitation of 761 mm^†^	Primary
NAM 38*			Saskatoon, Canada	Controlled greenhouse environment	Primary
*L. odemensis*	*L. ode.*	IG 72623	Latitude: 37.44 Longitude: 41.0167 (Midyat, Mardin, Turkey)	Hot, dry, and sunny summers, and cold, rainy, and snowy winters (Dalkılıç, 2012). Mean temperature ranges from 0.5°C in January to 35°C in June (Akgul et al., 2018). Mountainous terrain with an altitude of 1,100 m (Google Maps, 2021). Annual precipitation of 724 mm^†^	Secondary
*L. lamottei*	*L. lam.*	IG 110813	Latitude: 37.4167 Longitude: −4.25 (Lucena, Córdoba, Andalucía, Spain)	Hilly, forested terrain (Google Maps, 2021). Mediterranean climate with some continental features (Galán et al., 1995; Velasco-Jiménez et al., 2013). Hot, dry summers and cold, rainy winters with average temperature ranging from 7°C in winter to 30°C in summer (Galán et al., 1995). 660 m a.s.l. and annual precipitation of 644 mm^†^	Secondary
*L. ervoides*	*L. erv.*	L-01-827A	Single plant selection from PI 72847 from ICARDA^Φ^. For IG 72847 Latitude: 30.7667 Longitude: 35.6 (Ayn Al Bayda, Jordan)	Semi-arid climate with higher rainfall and lower evaporation rate in the winter, and mean temperature ranging from 9°C in the summer to 27°C in winter (Wade et al., 2010). Elevation of 1,250 m and annual precipitation of 304 mm^†^	Tertiary
*L. nigricans*	*L. nig.*	IG 116024	Latitude: 37.7667 Longitude: 29.1167 (Denizli, Turkey)	Same as PI 572376 but at elevation of 560 m a.s.l. and annual precipitation of 584 mm^†^	Quaternary

Gene pool classification is based on Wong et al. (2015). ^†^Plant genetic resources accession-level data provided by: International Center for Agricultural Research in the Dry Areas (ICARDA), http://www.icarda.org/, Lebanon and Morocco; and USDA National Plant Germplasm System, https://npgsweb.ars-grin.gov, United States. All intellectual property rights (including copyright) in the data are owned and retained by the said institutions. Data accessed through GENESYS Global Portal on Plant Genetic Resources, http://www.genesys-pgr.org, 2021-23-03. *Nested association mapping population 38. ^Φ^(Fiala et al., 2009).

The experiment was set up as a randomized complete block design with 12 genotypes and eight replicates. The chamber was set to 16-h day at 21°C, 8-h night at 15°C, and at ambient humidity. Light intensity ranged from 276 to 441 μmol m^–2^ s^–1^ depending on the height of the canopy and placement of the pot. Minimum temperature and maximum temperature during the day were 16.4 and 29.2°C, respectively, and the minimum temperature and maximum temperature during the night were 9.8 and 16.9°C, respectively. Plants were maintained under fully watered conditions for the duration of the study from January 26, 2018 to April 26, 2018.

The interspecific NAM 38 population was developed from the hybrid *L. cul.* CDC Redberry × *L. tom.* IG 72805, and single seed descent of 70 seeds was performed from the F2 population for four to six generations. The CDC Redberry parent seed was derived from the inbred seed source that was sequenced to produce the lentil reference genome available at the KnowPulse web portal (Vandenberg et al., 2006; Sanderson et al., 2019). There were 70 RILs of NAM 38 used, varying from the F_4_ to F_6_ generation of inbreeding. The NAM 38 lines were grown in the University of Saskatchewan Agriculture Greenhouse in Saskatoon, SK, Canada (52°08′21.3″N, 106°37′54.3″W) from October to December 2018. Temperature was set at 25°C/18°C day/night under a 16-h photoperiod. Temperature during the day ranged from 20.4 to 32.8°C, and the temperature during the night ranged from 16.2 to 21.8°C.

### Collection of Leaf Surface Imprints

**FIGURE 1:**
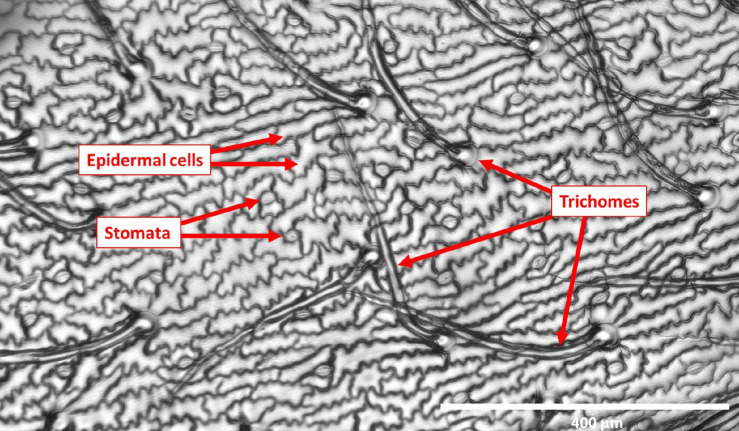
Representative image of a leaflet imprint at 10X magnification used to quantify micromorphological traits on the surface. Scale bar in image = 400 μm.

Imprints of leaf surfaces were made using SUMP disks and SUMP liquid (Sump Laboratory, Tokyo, Japan) as previously described in the study by Tanaka et al. (2005). At the early pod development (R3) stage (Erskine et al., 1990), three plants from each genotype were selected at random for leaf imprinting. Imprints of adaxial and abaxial surfaces of the youngest fully expanded leaflets were then made for a total of 36 plants belonging to the 12 genotypes. For the 70 individuals of the NAM 38 population, imprints were made only of the adaxial surface of the youngest fully expanded terminal leaflets. One leaflet imprint was made for each individual in the population. The impressions of leaflet surfaces were visualized and analyzed with the aid of an EVOS FL inverted microscope (Mill Creek, Washington, United States). Three fields of view were captured for each leaflet sample for both adaxial and abaxial surfaces. Scale for each image was automatically generated by the microscope at the time of visualization. The data obtained for each image were TD, TL, stomatal density (SD), and epidermal cell density (ECD). In a selected area of leaf surface, the number of trichomes, stomata, and epidermal cells was counted manually and subsequently measured using ImageJ software^1^. Figure 1 shows a representative imprint image used to quantify micromorphological surface traits.

Trichome length was measured by tracing along the trichomes and using the “Length” measurement on ImageJ using the calibrated scale bar on the images. SIs were then calculated for each genotype using the formula introduced by Salisbury and Oliver (1928):

$$\begin{array}{l} Stomatal\;index\;(\%)\\ =\frac{Number\;of\;stomata\;per\;unit\;area}{Number\;of\;stomata\;+\;Number\;of\;epidermal\;cells\;per\;unit\;area}\times 100 \end{array}$$

### Scanning Electron Microscopy

Scanning electron microscopy was conducted to visualize the qualitative differences in morphology and topography among leaflets and pods of the 12 lentil genotypes. The youngest fully expanded leaflets of the rachis were sampled. Pods were sampled at the R4 (flat pod) stage (Erskine et al., 1990). Leaf and pod samples were fixed in 100% ethanol at 4°C for at least 24 h. The samples were then subjected to critical point drying using CO_2_ at approximately 31.5°C and 1200 psi (lb/in^2^) in a Polaron E3000 Critical Point Dryer (Quorum Technologies Ltd., East Sussex, United Kingdom), then gold-coated in Edwards S150B Sputter Coater (BOC Edwards, United Kingdom), and examined using a Phenom G2 pure desktop SEM (Phenom-World, Eindhoven, Netherlands). Leaflet and pod samples were imaged at 180–185X magnification in Topographic mode for leaflets and normal (full) mode for pods.

### Data Analyses

Data for TD, TL, ECD, SD, and SI were fitted using a linear mixed model with the interaction between surface and genotype as a fixed variable and blocking as the random variable. All analyses were done using R Statistical Software (R Core Team, 2019), and significant differences in response variables were calculated using the least-square means method for multiple comparisons at alpha = 5% and adjusting *p*-value using the Tukey’s method after ensuring homogeneity of variances through Levene’s test from the package “car” (Fox and Weisberg, 2019). The lme function from the package lme4 was used to fit the model, and the function lsmeans from the package emmeans was used to calculate the least-square means (Bates et al., 2015; Lenth, 2019). Data were plotted using SigmaPlot version 11.0 (Systat Software, San Jose, CA, United States).

## Results

### Characterizing Trichomes in Lentil Genotypes

**FIGURE 2:**
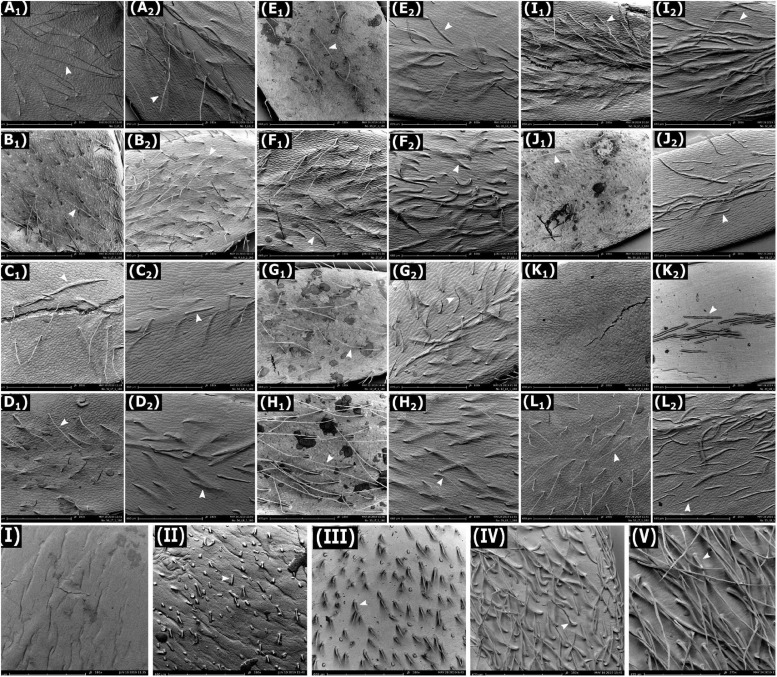
Scanning electron microscopy images of adaxial (indicated as subscript 1) and abaxial (indicated as subscript 2) surfaces of leaflets of lentil genotypes. **(A)** *L. cul.* Indianhead and **(B)** *L. cul.* CDC Redberry at 185X magnification and the following at 180X magnification: **(C)** *L. cul.* CDC Greenstar, **(D)** *L. ori.* IG 72643, **(E)** *L. ori.* PI 572376, **(F)** *L. tom.* IG 72613, **(G)** *L. tom.* IG 72614, **(H)** *L. tom.* IG 72805, **(I)** *L. ode.* IG 72623, **(J)** *L. lam.* IG 110813, **(K)** *L. erv.* L-01-827A, and **(L)** *L. nig.* IG 116024. Images **(I–V)** are surfaces of lentil pods at 180X magnification for **(I)** *L. cul.* Indianhead, **(II)** *L. cul.* CDC Redberry, **(III)** *L. erv.* L-01-827A, **(IV)** *L. tom.* IG 72613, and **(V)** *L. tom.* IG 72805. Arrows indicate trichomes. Images **(K_1_)** and **(I)** lack trichomes.

Scanning electron microscopy revealed that trichomes of all lentil genotypes were simple, unicellular, tapered, non-glandular hair-like structures (Figures 2A–L). Distribution of trichomes was even across the entire leaflet surface in all genotypes except *L. lam.* IG 110813 and *L. erv.* L-01-827A, where more trichomes were clustered along the midrib compared to other areas. Trichomes on pods were categorized into five phenotype classes based on visual observations: no trichomes or glabrous pods (*L. cul.* Indianhead), very short trichomes at low density (*L. cul.* CDC Redberry), short trichomes at high density (*L. erv.* L-01-827A), medium-length trichomes at high density (*L. tom.* IG 72613), and long trichomes at high density (*L. tom.* IG 72805) (Figures 2I–V, respectively).

**FIGURE 3:**
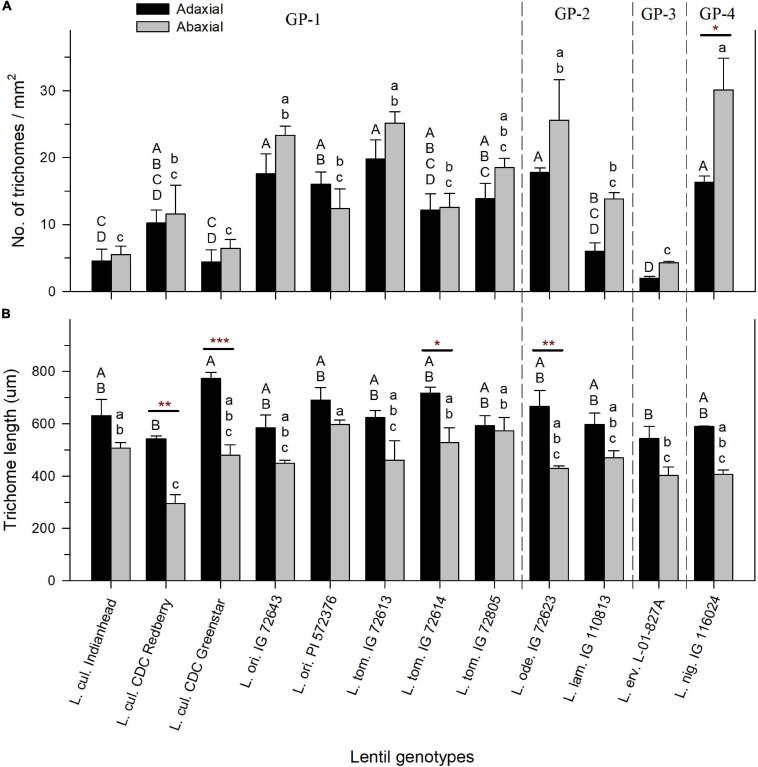
Mean trichome density expressed as the number of trichomes per mm^2^ **(A)** and mean trichome length in micrometers **(B)** on the adaxial and abaxial leaflet surfaces of cultivated and wild lentil genotypes. Asterisks indicate statistical difference between mean density of adaxial and abaxial surfaces of the same genotype based on *t*-test (Significance: *p < 0.05; **p < 0.01; ***p < 0.001). No asterisk implies no significant difference. Uppercase and lowercase letters show significant differences between adaxial and abaxial surfaces, respectively, across all genotypes at α = 5%. GP-1, GP-2, and GP-3 denote the genotypes belonging to primary, secondary, and tertiary gene pools, respectively, based on Wong et al. (2015).

A large range of variation in leaflet TD was observed across wild and cultivated lentil genotypes. TD on adaxial leaflet surfaces ranged from 2 (±0.2) trichomes/mm^2^ in *L. erv.* L-01-827A to 20 (±2.8) trichomes/mm^2^ in *L. tom.* IG 72613 (Figure 3A). On the abaxial leaflet surface, TD ranged from 4 (±0.2) trichomes/mm^2^ in *L. erv.* L-01-827A to 30 (±4.8) trichomes/mm^2^ in *L. nig.* IG 116024 (Figure 3A). All lentil genotypes had increased TD on the abaxial leaflet surface, except for *L. ori.* PI 572376, which had a higher TD on the adaxial leaflet surface. TD between the adaxial and abaxial leaflet surfaces of the same genotype did not differ significantly except in the case of *L. nig.* IG 116024, where there were 1.8 times more trichomes on the abaxial surface compared to the adaxial surface.

Trichomes on the adaxial surface were consistently longer compared to those on the abaxial surface in lentil genotypes across all species. On the adaxial surface, the longest TL was observed in *L. cul*. CDC Greenstar (773 ± 24 μm), and the shortest TL was observed in *L. cul*. CDC Redberry (543 ± 11 μm) (Figure 3B). On the abaxial surface, trichomes of *L. ori*. PI 572376 (597 ± 17 μm) and *L. cul*. CDC Redberry (295 ± 35 μm) were the longest and shortest, respectively (Figure 3B). Difference in TL between adaxial and abaxial surface within each genotype was in most cases non-significant, except for the following four genotypes that had significantly longer trichomes on the adaxial surface compared to the abaxial surface: *L. cul.* CDC Redberry, *L. cul.* CDC Greenstar, *L. tom.* IG 72614, and *L. ode.* IG 72623.

### Characterizing Stomatal and Epidermal Cell Traits in Lentil Genotypes

To determine the type of stomata, cells in contact with the guard cells were only considered subsidiary cell(s) if they appeared to be distinctly different from the neighboring pavement cells in shape, size, or morphology (Gray et al., 2020). All genotypes had anomocytic stomata (Table 2). Additionally, several genotypes had a stomatal complex where one cell in contact with the stomata was significantly smaller than the rest of the neighboring cells and was parallel to the lateral axis of the stomatal pore. These were considered hemi-paracytic stomata as per the definition by Van Cotthem (1970). In all genotypes, stomata on the adaxial surface were slightly sunken below the plane of the epidermal cells, while stomata on the abaxial surface were on the same plane as the epidermal cells.

**TABLE 2:** Qualitative stomatal and epidermal cell traits in 12 wild and cultivated lentil genotypes.

**Species**	**Surface**	**Type(s) of stomata present**	**Anticlinal wall pattern in epidermal cells**
*L. cul.* Indianhead	Adaxial	Anomocytic	Sinuous
	Abaxial	Anomocytic	Sinuous
*L. cul*. CDC Redberry	Adaxial	Anomocytic, Hemi-paracytic	Sinuous
	Abaxial	Anomocytic	Sinuous
*L. cul*. CDC Greenstar	Adaxial	Anomocytic, Hemi-paracytic	Sinuous
	Abaxial	Anomocytic	Sinuous
*L. ori.* IG 72643	Adaxial	Anomocytic, Hemi-paracytic	Sinuous
	Abaxial	Anomocytic, Hemi-paracytic	Sinuous
*L. ori*. PI 572376	Adaxial	Anomocytic, Hemi-paracytic	Sinuous
	Abaxial	Anomocytic, Hemi-paracytic	Sinuous
*L. tom*. IG 72613	Adaxial	Anomocytic, Hemi-paracytic	Sinuous
	Abaxial	Anomocytic, Hemi-paracytic	Sinuous
*L. tom*. IG 72614	Adaxial	Anomocytic, Hemi-paracytic	Sinuous
	Abaxial	Anomocytic, Hemi-paracytic	Sinuous
*L. tom*. IG 72805	Adaxial	Anomocytic, Hemi-paracytic	Sinuous
	Abaxial	Anomocytic, Hemi-paracytic	Sinuous
*L. ode*. IG 72623	Adaxial	Anomocytic, Hemi-paracytic	Sinuous
	Abaxial	Anomocytic, Hemi-paracytic	Sinuous
*L. lam*. IG 110813	Adaxial	Anomocytic	Undulate
	Abaxial	Anomocytic, Hemi-paracytic	Sinuous
*L. erv*. L01827A	Adaxial	Anomocytic, Hemi-paracytic	Sinuous
	Abaxial	Anomocytic, Hemi-paracytic	Sinuous
*L. nig*. IG 116024	Adaxial	Anomocytic	Sinuous
	Abaxial	Anomocytic, Hemi-paracytic	Sinuous

All genotypes had irregularly shaped epidermal cells. Pattern on the epidermal cell wall was observed for both surfaces of each genotype based on schematic images by Nishida and Christophel (1999). Anticlinal walls of the epidermal cells in most species had a sinuous outline, except for the adaxial surface of *L. lam*. 110813, which was undulated with fewer curves than other genotypes (Table 2). The degree of convolution of anticlinal walls varied between surfaces: Anticlinal walls of epidermal cells on the abaxial surface were more convoluted than the ones on the adaxial surface.

**FIGURE 4:**
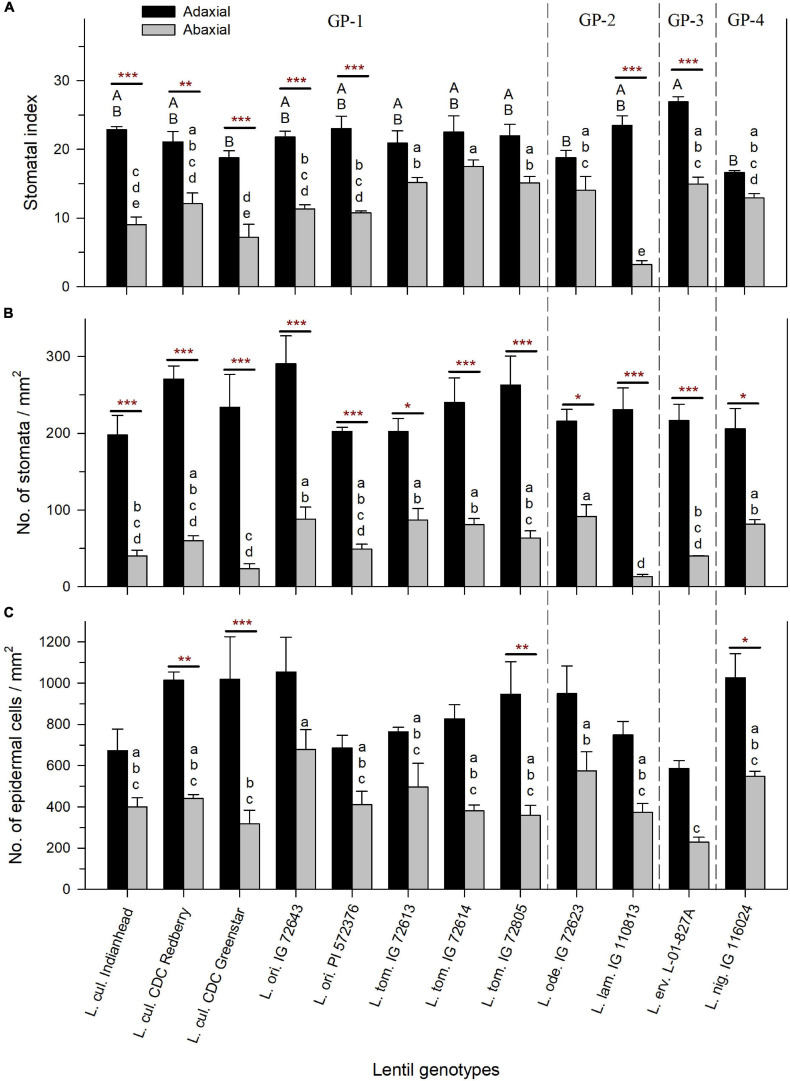
Stomatal index expressed as % **(A)**, stomatal density expressed as the number of stomatal pores per mm^2^ **(B)**, and epidermal cell density expressed as the number of epidermal cells per mm^2^ **(C)** on the adaxial and abaxial leaflet surface of cultivated and wild lentil genotypes. Stars represent the statistical difference between adaxial and abaxial surfaces of the same genotype based on *t*-test (Significance: *p < 0.05; **p < 0.01; ***p < 0.001). No stars imply no significant difference. Uppercase and lowercase letters show significant differences between the adaxial and abaxial surfaces, respectively, across all genotypes at alpha equals 5%. Absence of letters implies no significant difference. GP-1, GP-2, and GP-3 denote the genotypes from the primary, secondary, and tertiary gene pools, respectively (Wong et al., 2015).

Stomatal index was influenced by SD and ECD. SI, SD, and ECD were consistently higher on the adaxial leaf surface compared to the abaxial surface across all species (Figures 4A–C). SI on the adaxial surface ranged from 17% (±0.3%) in *L. nig*. IG 116024 to 27% (±0.8%) in *L. erv.* L-01-827A, and SI on the abaxial surface ranged from 3% (±0.6%) in *L. lam*. IG 110813 to 18% (±0.9%) in *L. tom*. IG 72614 (Figure 4A). There was a significant difference in SI between adaxial and abaxial surfaces in all species except for *L. tom.*, *L. ode.*, and *L. nig.*

Stomatal density was calculated by counting the number of stomatal pores per square mm on both surfaces. SD on the adaxial surface was not significantly different between genotypes and ranged from 198 (±25) stomata/mm^2^ (in *L. cul*. Indianhead) to 291 (±36) stomata/mm^2^ (in *L. ori*. IG 72643) (Figure 4B). On the abaxial surface, SD varied significantly between genotypes with *L. lam.* IG 110813 having the lowest SD of 13 (±3) stomata/mm^2^ and *L. ode.* IG 72623 having the highest SD of 92 (±15) stomata/mm^2^. SD in all wild and cultivated genotypes was significantly higher on the adaxial surface compared to the abaxial surface.

Epidermal cell density followed a similar trend as SD. ECD on the adaxial surface was not significantly different among genotypes and ranged from 585 (±40) epidermal cells/mm^2^ (in *L. erv.* L-01-827A) to 1055 (±167) epidermal cells/mm^2^ (in *L. ori.* IG 72643) (Figure 4C). On the abaxial surface, ECD was lower and varied from 230 (±23) epidermal cells/mm^2^ in *L. erv.* L-01-827A to 679 (±95) epidermal cells/mm^2^ in *L. ori.* IG 72643. For most genotypes, ECD on the adaxial and abaxial surfaces did not differ significantly. However, the following genotypes had a significant decrease in ECD on their abaxial surface compared to their adaxial surface: *L. cul.* CDC Redberry (2.3X difference), *L. cul.* CDC Greenstar (3.2X difference), *L. tom.* IG 72805 (2.6X difference), and *L. nig.* IG 116024 (1.9X difference).

### Interaction of Morphological Traits in 12 Lentil Genotypes

Combined analysis of quantitative morphological traits of 12 lentil genotypes *via* two-factor ANOVA showed significant effects of genotype and leaflet surface characteristics for all traits, while the effects of the interaction between genotype and surface were only significant for the TL and SI (Table 3). Effect of blocking was only significant for TL and was insignificant for all other traits.

**TABLE 3:** Summary of the effects of genotype, adaxial and abaxial leaflet surfaces, and their interaction on micromorphological leaflet traits in wild and cultivated lentil.

	**Genotype**		**Surface**		**Genotype × Surface**	
**Variables**	***F*-value**	***P*-value**	***F*-value**	***P*-value**	***F*-value**	***P*-value**
TD (mm^–2^)	16.81	<0.001	16.35	<0.001	1.71	ns
TL (μm)	6.23	<0.001	138.99	<0.001	2.38	0.022
SI (mm^–2^)	7.26	<0.001	348.96	<0.001	7.18	<0.001
SD (mm^–2^)	2.25	0.030	441.69	<0.001	1.75	ns
ECD (mm^–2^)	4.21	<0.001	145.13	<0.001	1.29	ns

*TD, trichome density; TL, trichome length; SI, stomatal index; SD, stomatal density; ECD, epidermal cell density; ns, not significant (*p*-value > 0.05). *F*-values are obtained after conducting two-factor ANOVA.*

**FIGURE 5:**
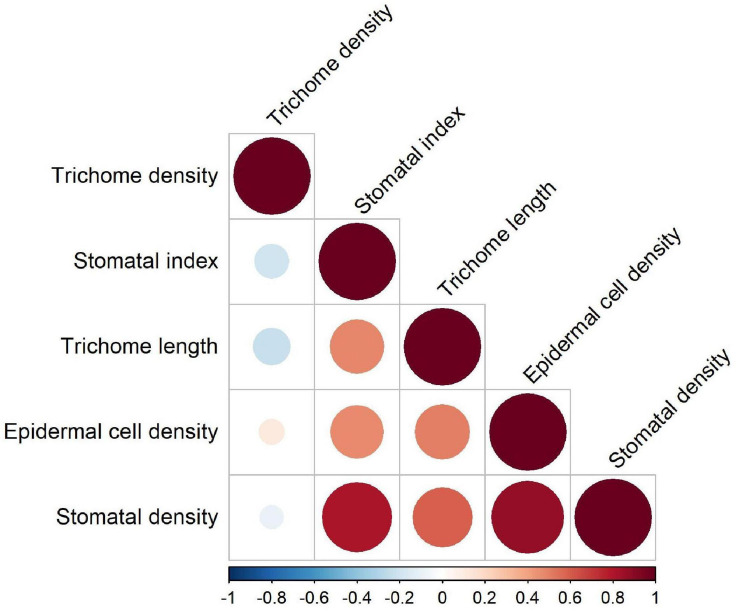
Correlations between five micromorphological traits on the adaxial and abaxial leaflet surfaces of wild and cultivated lentil.

Combined correlation analysis for 12 lentil genotypes revealed that TD was negatively correlated with SI, TL, and SD (Figure 5). These negative correlations were not statistically significant and had *p*-values > 0.05. TL was significantly positively correlated with SI, SD, and ECD. Since SI is a function of stomatal and ECD, all three were significantly positively correlated with each other. For genotypes belonging to wild species, precipitation and altitude at center of origin were not strongly correlated with any trait (correlation coefficient = 0 to 0.35 for positive correlations and −0.28 to 0 for negative correlations).

### Trichome Density and TL in an Interspecific Hybrid Population of *L. culinaris* × *L. tomentosus*

**FIGURE 6:**
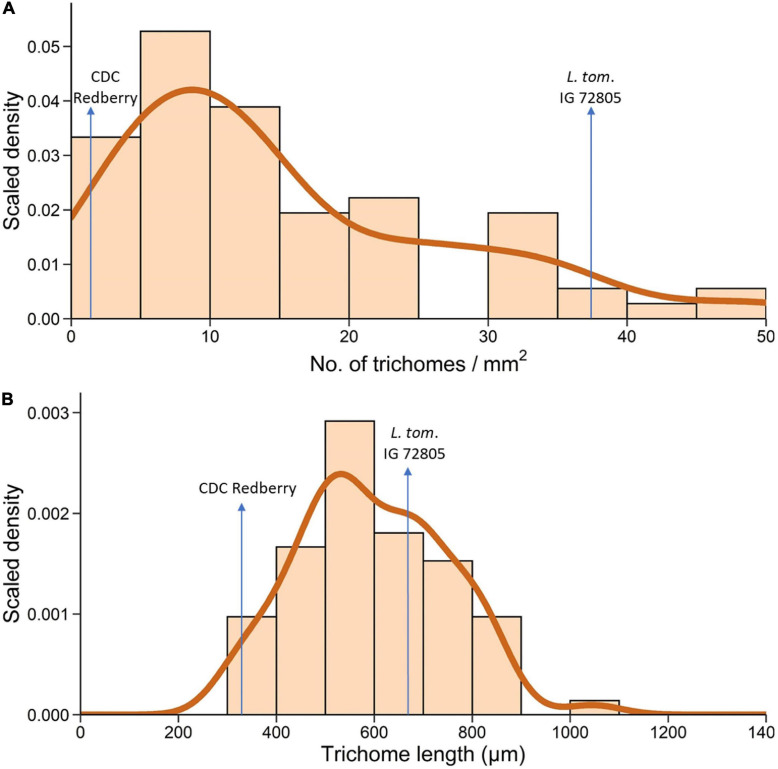
Histogram and density plots of trichome density **(A)** and trichome length **(B)** on the adaxial leaflet surface of 70 RILs of *L. cul.* × *L. tom.* NAM 38 varying from F_4_ to F_6_ generation.

A total of 70 RILs of the NAM 38 population obtained from crossing *L. cul*. CDC Redberry with *L. tom*. IG 72805 were assessed for TD and TL on the adaxial leaf surface. These RILs varied from F_4_ to F_6_ generation. Histogram and density plots revealed non-symmetric skewed distributions for both traits (Figure 6). In the NAM 38 population, TD ranged from 1.11 mm^–2^ to 50 mm^–2^ with the density curve peaking around 10 mm^–2^ and skewing toward the right (Figure 6A). For reference, TD of cultivated and wild parents of NAM 38 was 1.8 mm^–2^ and 37.5 mm^–2^, respectively. Trichome characteristics were observed to be segregating transgressively in the RIL population.

The histogram and the resulting density plot for TL were almost symmetric with a slight skew toward the right. TL in the RILs ranged from 314 μm to 1,046 μm with the density curve peaking around 500 μm and a smaller peak around 700 μm (Figure 6B). TL of NAM 38 parents was 332 μm (*L. cul.* CDC Redberry) and 670 μm (*L. tom.* IG 72805).

## Discussion

Lentil germplasm exhibits a wide range of variation in surface microstructures of trichomes, stomata, and epidermal cells on its foliar and pod surfaces. Even though trichome type (simple, non-glandular, and unicellular hair-like structures) was uniform across wild and cultivated lentil species, their density and length varied across species. Our results verify the limited amount of available literature on this topic: Variation in the pubescence of cultivated lentil is documented and there are records that describe lentil cultivars and landraces from Spain, the Indian subcontinent, and the western Mediterranean region as having absent, slight, or dense leaf pubescence (Lázaro et al., 2001; Toklu et al., 2009; Kumar and Solanki, 2014). A comprehensive study by Singh et al. (2014) that described various lentil traits across accessions belonging to all seven lentil species also characterized leaf pubescence as absent, slight, or dense. Thus far, leaf pubescence in lentil has only been described as a qualitative trait based on trait descriptors set by IBPGR/ICARDA (IBPGR and ICARDA, 1985). By quantifying TD, our study expands on this information and highlights the variation in leaf pubescence, not only among different species, but for the first time, also on abaxial and adaxial surfaces within the same genotype.

Our study also revealed various types of pubescence characteristics on pod surfaces. Ferguson et al. (2000) stated that *L. erv.* can be distinguished from other wild species due to its puberulent pods. We confirmed this in our study (Figure 2III) and found that no other species exhibited this phenotype on its pods. In contrast, however, Ferguson et al. (2000) noted *L. cul.* to have glabrous pods. We found that sparse trichomes may occur in *L. cul.*, for example in CDC Redberry. Leaflet TD in CDC Redberry was also higher than other *L. cul.* genotypes. Since CDC Redberry is resistant to fungal diseases of ascochyta blight and race 1 of anthracnose (Vandenberg et al., 2006), it is possible that the trait of trichomes contributes to this combined disease resistance and needs to be further investigated. Differences in pubescence were also observed on stems of the different species as corroborated by Hoque et al. (2002), but were not characterized in this study.

Trichome density in genotypes (calculated by taking an average of TD on adaxial and abaxial surface of each genotype) ranged from 3 trichomes/mm^2^ (in *L. erv.* L-01-827A) to 23 trichomes/mm^2^ (in *L. nig.* IG 116024). TD in other wild and cultivated genotypes varied between these two extremes. Even though no correlation was found between trichome traits and the altitude and precipitation at the center of origin of wild species, it is possible that the differences in TD are due to the allopatric distribution of different lentil species: Presumably, they differentiated genetically and morphologically over time in unique environments. Trichomes play an important role in mediating plant–environment interactions such as defense against biotic and abiotic stressors like UV radiation, herbivores, and pathogens (Karabourniotis et al., 2020), so it is possible that they evolved over time in response to unique stress combinations encountered by each species at their respective centers of origin. Interestingly, drastic differences in TD were also present between genotypes belonging to the same species, even when their centers of origin were the same. For example, even though *L. tom.* IG 72613 and *L. tom.* IG 72614 have the same centers of origin, TD on surfaces of *L. tom.* IG 72614 was half that of *L. tom.* IG 72613. While this difference between the two *L. tom.* genotypes was statistically insignificant for either of the adaxial or of the abaxial surfaces, more research is needed to explain the intraspecific variation in TD observed in this study.

Most genotypes had lower TD on their adaxial leaflet surface compared to the abaxial leaflet surface, even though these differences were statistically non-significant. This phenomenon has been observed in other plants adapted to the Mediterranean climate, and it has been shown that the abaxial surface with higher TD is more effective than the adaxial surface at attenuating UV-A and UV-B radiation and protecting developing plants against high visible irradiance (Karabourniotis and Bornman, 1999). Additionally, while TD was generally higher on the abaxial surface, trichomes were shorter on the abaxial surface of all genotypes. This suggests that there might be a compensation between length and density, possibly related to the effect of changes in adaptation to the environment over time. Concurrent with the reduced TD on the adaxial surface, genotypes of all wild and cultivated species had a higher density of slightly sunken stomata on the adaxial surface. Sunken stomata were not observed on the abaxial surface of any genotype. Sunken stomata are a xeromorphic feature aimed at reducing transpiration (Haworth and McElwain, 2008), and it may be that the sunken stomata trait along with longer trichomes in wild and cultivated lentil is an adaptation to conserve water and compensate for reduced trichome cover on the adaxial surface.

Stomatal index was similar when comparing the same leaf surface across all species, but was higher on the adaxial surface compared to the abaxial surface within genotypes (Figure 4A). The same trend was observed for SD and ECD. These results are consistent with those observed by Sánchez-Gómez et al. (2019) in three Spanish lentil cultivars, and those of Lopes and Veiga (1979) who assessed cultivated lentil varieties and lines from the United States, Canada, Chile, and Brazil. However, the latter study observed that accessions with the highest SI on the adaxial surface also had the lowest SI on the abaxial surface. This suggests that there is compensation between the two surfaces with respect to this trait (Lopes and Veiga, 1979). Our study did not support this finding within the same gene pool or species, except in the case of *L. ori.* PI 572376, which had the highest and lowest SI on the adaxial and abaxial surface, respectively. These observations suggest that each genotype is unique in its physiology and that all lentil genotypes do not regulate their surface microstructural properties in the same manner. Additionally, the higher density of stomata on the adaxial surface in lentil is in contrast to most other amphistomatic plants, which usually have increased SD on the abaxial surface (Xiong and Flexas, 2020). Higher SD on the adaxial surface has been reported, however, in a C_3_ monocot grass species by Anderson and Briske (1990), but they noticed that this species had a 180° twist at its leaf blade which oriented the adaxial surface of leaves to match the orientation of other grasses that had higher stomata on their abaxial surface. It is not known if *Lens* spp. exhibits such a phenotype as well. It must be noted here that while amphistomatous plants may have an advantage over hypostomatous plants in terms of having higher gas exchange capacity (Drake et al., 2019), it is unclear if stomatal pore efficiency differs between the two surfaces, and thus, further investigation is needed to elucidate the advantages and disadvantages of amphistomaty, particularly the presence of stomata on the adaxial surface (Drake et al., 2019; Xiong and Flexas, 2020).

The types of stomata and anticlinal wall pattern of epidermal cells in *Lens* spp. were also determined for the first time *via* this study. All wild and cultivated genotypes had anomocytic stomata, and most also contained hemi-paracytic stomata, characterized by having one cell in the stomatal complex that was significantly smaller than other neighboring cells and was parallel to the lateral axis of the stomatal pore. It must be noted that in this study, we considered only those cells to be subsidiary cells that appeared distinct from other neighboring cells. Since most cells surrounding the guard cells appeared similar to other cells on the epidermis in shape and size, most stomata were classified as anomocytic. Anticlinal walls of epidermal cells on both adaxial and abaxial surfaces were sinuous in all genotypes except for the adaxial surface of *L. lam.* 110813. The phenotype of amphistomatic leaves, presence of anomocytic stomata, and adaxial and abaxial surfaces possessing different types of epidermal anticlinal wall patterns have also been reported in some other legumes such as lotus (Stenglein et al., 2003) and faba bean (Ahmad et al., 2010).

After quantifying trichome, stomatal, and epidermal cell traits in *Lens* spp., we sought to explore the inheritance of trichome traits. Previous studies of lentil pubescence within the cultivated species determined that pubescence is a qualitative trait controlled by a single gene system for which pubescence is dominant to glabrous phenotype in pods, peduncle, and the whole plant (Vandenberg and Slinkard, 1989; Sarker et al., 1999; Hoque et al., 2002). These studies did not go beyond the F_2_ or F_3_ generation, and the presence or absence of pubescence was determined through the visual inspection. In our study, after quantifying leaflet TD in the interspecific *L. cul.* × *L. tom.* NAM 38 RILs at F_4_-F_6_ generation, we found that plants segregated transgressively for TD and had an almost continuous distribution of TD where 45 out of 70 lines had low-medium TD (<15 mm^–2^) (Figure 6A). Transgressive segregation was also observed for TL and the distribution resembled a bell-shaped curve (Figure 6B). These combined results based on NAM 38 RILs suggest that leaflet pubescence is a quantitative trait or that the alleles of the genes that control plant pubescence are responsible for tissue-specific expression, as speculated by Kumar et al. (2005). This information will be useful from an agronomic perspective for plant breeders wanting to breed the trait of trichomes into cultivated lentil for sustained biotic and abiotic stress tolerance.

## Conclusion

Our study highlighted the presence of a wide range of diversity in trichome and stomatal characteristics across the lentil germplasm. Trichome variation observed in wild and cultivated genotypes was not consistent across gene pools or even among lentil genotypes belonging to the same species, suggesting that the regulation of morphophysiological traits of trichomes is uniquely different in each genotype and likely evolved as a response to unique stresses in their centers of origin. Despite these differences, certain trends were observed in the traits of SD, trichomes, and ECD as they are related to the differences between adaxial and abaxial surfaces across the lentil germplasm. TL, SI, SD, and ECD were higher on the adaxial surface compared to the abaxial surface in each genotype, and these traits were significantly correlated with each other. TD was negatively correlated with SI and TL, suggesting that there may be a trade-off between these traits. The adaxial surface of all lentil genotypes, despite having more stomata, exhibited slightly sunken stomata, which might be a xeromorphic adaptation.

The study of interspecific RILs of *L. cul.* CDC Redberry and *L. tom*. IG 72805 revealed that TD and TL segregate transgressively. Quantifying and plotting these traits resulted in asymmetric density plots of continuous data, suggesting that the traits of TD and TL are quantitatively inherited. There is potential for harnessing the diversity in pubescence and stomatal traits to create future lentil varieties with desirable characteristics that can contribute to better adaptation to the future challenges of biotic and abiotic stresses.

Species delimitation based on the traits explored in this study alone is not possible since there were not any morphological or anatomical traits of trichomes, stomata, or epidermal cells that were unique to a particular species. However, these are important agronomic morphophysiological traits that have been shown to play a key role in mediating plant responses to biotic and abiotic stresses. TD and TL appear to be quantitatively inherited. If any of these traits are identified as being advantageous for lentil in imparting resistance or tolerance to biotic or abiotic stresses, then the results from this study will help physiologists and breeders make better decisions in selecting species or genotypes that better fit their goal of breeding lentil with improved resistance to concurrent stresses.

## Data Availability Statement

The raw data supporting the conclusions of this article will be made available by the authors, without undue reservation.

## Author Contributions

IP, LG, KT, and AV contributed to the conception, design, and planning of the experiments. IP performed the experiments, analyzed the data, and wrote the manuscript with input from LG. KT and AV revised the manuscript and approved the submitted version. All authors contributed to the article and approved the submitted version.

## Conflict of Interest

The authors declare that the research was conducted in the absence of any commercial or financial relationships that could be construed as a potential conflict of interest.

## Footnotes

1 imagej.nih.gov/ij/
